# Clinicopathological Characteristics, Treatment, and Prognosis of 21 Patients with Primary Gastric Squamous Cell Carcinoma

**DOI:** 10.1155/2016/3062547

**Published:** 2016-07-05

**Authors:** Yang Chen, Hong Zhu, Feng Xu, Yidan Cao, Xingting Gu, Yuming Wan, Hongfeng Gou

**Affiliations:** Cancer Center, West China Hospital, Sichuan University, 37 Guoxue Alley, Chengdu, Sichuan 610041, China

## Abstract

We performed a retrospective analysis of 21 patients with primary gastric squamous cell carcinoma (PGSCC) who were admitted to our hospital from October 2008 to October 2014. The median age was 67 years and male predominance was observed, the most common tumor locations were the upper third of the stomach, most of the clinical manifestations were identical to those of other types of gastric tumors, and the tumor cells had positive immunoreactivity for p63 and CK5/6. In terms of treatments, surgery (R0 resection) is the main treatment; the effect of other treatments is unclear. The median survival time for the surgery group and nonsurgery group was 46 and 4.5 months, respectively. Probably due to limited number of cases, no significant difference in median survival time was observed between the surgery alone group and the surgery plus adjuvant therapy group (46 versus 51 months, *P* = 0.310). A standard chemotherapy regimen for this disease has not yet been established; the choice of its chemotherapy regimens tends to follow the principle of the treatment of gastric adenocarcinoma or esophageal cancer. PGSCC generally had a poor prognosis, and early detection, early diagnosis, and early surgical treatment are beneficial to patients.

## 1. Introduction

Gastric cancer is one of the most common malignancies and a frequent cause of cancer-related death in the world, especially in China. Among gastric cancers, adenocarcinoma is the most prevalent type and accounts for more than 90% of gastric cancers, but primary gastric squamous cell carcinoma (PGSCC) is extremely rare, accounting for about 0.04% to 0.07% of the total gastric carcinomas worldwide [1, 2]. Due to its rarity, reports on it were limited, PGSCC was first identified in 1895, and less than 100 cases of PGSCC have been reported and all of the published data are of solitary cases. Although theories exist regarding its development in the stomach, the pathogenesis of this tumor has not been well elucidated. The optimal treatment strategy is controversial. PGSCC is often diagnosed at a late stage and its prognosis is generally poor. Our understanding of PGSCC remains poor; in order to contribute to a deeper knowledge of this tumor, we performed a retrospective analysis of the clinicopathological characteristics, treatment, and prognosis of 21 patients with PGSCC who were admitted to our hospital from October 2008 to October 2014. To our best knowledge, our study included the largest series of patients with PGSCC analyzed to date.

## 2. Materials and Methods

### 2.1. Patients Selection

Patients with pathologically proven gastric carcinoma were screened for enrollment in the study. The pathological specimens were endoscopic biopsied or resected specimens from surgery. Inclusion criteria were pathologically proven squamous cell carcinoma and no evidence of SCC in any part of the body diagnosed between October 2008 and October 2014 with available medical records and follow-up data.

### 2.2. Data Collection and Follow-Up

The demographic data, clinical manifestation at the time of diagnosis, the performance status (PS) score, tumor's size and location, values of hemoglobin, albumin, and serum calcium on admission, pathological data (Borrmann types, depth of invasion, metastasis of regional lymph nodes, and immunohistochemical staining), TNM stages, and treatment were extracted from medical records and pathological reports. Contrast-enhanced computed tomography scan of the chest and abdomen, electronic gastroscopy, blood routine examination, biochemical test, and tumor serum markers detection were routinely performed. TNM stages were reviewed according to the criteria of the Seventh Edition of the American Joint Committee on Cancer Staging Manual [3]. The PS score was evaluated according to the Eastern Cooperative Oncology Group (ECOG). The follow-up was done by phone calls, reviewing the patients' medical records, or periodic outpatient follow-up; follow-up was ended in November 2015. The untreated patients were included in the statistical analysis.

### 2.3. Statistical Analysis

The SPSS software program (version 20.0; IBM Corporation) was used for statistical analysis, the Kaplan-Meier method was used for the survival analysis, and survival curves were plotted; a log-rank test was used for comparison of significance between different survival curves. *P* < 0.05 was set as statistical significance. Survival time was calculated from the time of diagnosis to death or until the last follow-up.

## 3. Results

### 3.1. Patient Characteristics

The clinicopathological data of the 21 PGSCC patients are summarized in Tables 1 and 2. The research objects were composed of 18 males (85.7%) and 3 females (14.3%), with a male-female ratio of 6 : 1. The median age was 67 years (range, 48–75 years), 13 males had a history of smoking, and the years of smoking were all more than ten years, while the rest of 8 patients never smoked. There were 17 cases (81%) with a PS score of 0 or 1, and 4 cases (19%) with a PS score above 2. The most common tumor locations were the upper third of the stomach (66.7%), followed by the middle third (28.6%) and the lower third (4.7%), with a median size of 5.8 cm (range: 3 cm to 15 cm). Borrmann types I, II, and III carcinomas were observed in 9 (42.8%), 6 (28.6%), and 6 (28.6%) cases, respectively. The clinical manifestations included 14 cases of abdominal pain (66.7%), 10 cases of dysphagia (47.6%), 8 cases of nausea and vomiting (38%), 7 cases of melena or hematochezia (33.3%), 6 cases of haematemesis (28.6%), and 4 cases of obviously weight loss recently (19%).

### 3.2. Hematologic Studies Characteristics

Laboratory values on admission including blood routine examination, biochemical tests, and tumor serum markers detection were routinely performed in 21 patients with PGSCC. For blood routine examination, 14 patients (66.7%) had anemia; mild, moderate, and severe anemia were found in 8 (38%), 3 (14.3%), and 2 (14.4%) cases, respectively. For biochemical detection, hypoalbuminemia and hypocalcemia were found in 9 (42.9%) and 9 (42.9%) cases, respectively. For tumor serum markers tests, elevated values of CEA and CA19-9 were found in 8 (38%) and 7 (33.3%) cases, respectively.

### 3.3. Pathological and Immunohistochemical Examination

**Figure 1:**
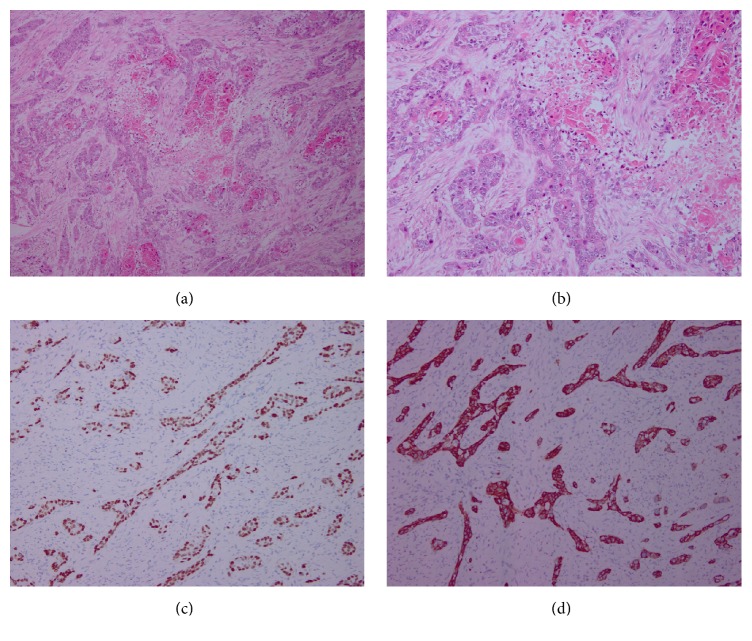
Pathological and immunohistochemical characteristics of squamous cell carcinoma ((a) hematoxylin-eosin staining, ×100; (b) hematoxylin-eosin staining, ×400); immunohistochemistry of p63 ((c) ×40) and CK5/6 protein ((d) ×100) that was positive in the squamous cell carcinoma area.

For pathological examination, most squamous cell carcinoma components had the characteristics of individual cell keratinization, keratin pearl, or intercellular bridge, among others. Four patients in our study underwent immunohistochemical examination; the tumor cells had positive immunoreactivity for p63 and CK5/6 (Figure 1).

### 3.4. Treatment and Prognosis

**Figure 2:**
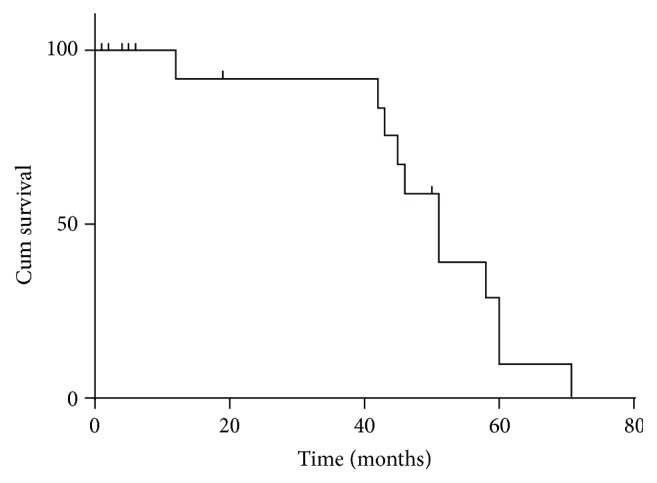
Survival curve for 21 patients with PGSCC.

The patients were divided into two groups based on treatment subtype as follows: fifteen patients underwent curative surgery (surgery group); six received no surgery (nonsurgery group). The median survival time for the surgery group and nonsurgery group was 46 and 4.5 months, respectively. Survival curve for 21 patients with PGSCC is plotted (Figure 2).

### 3.5. The Surgery Group

**Figure 3:**
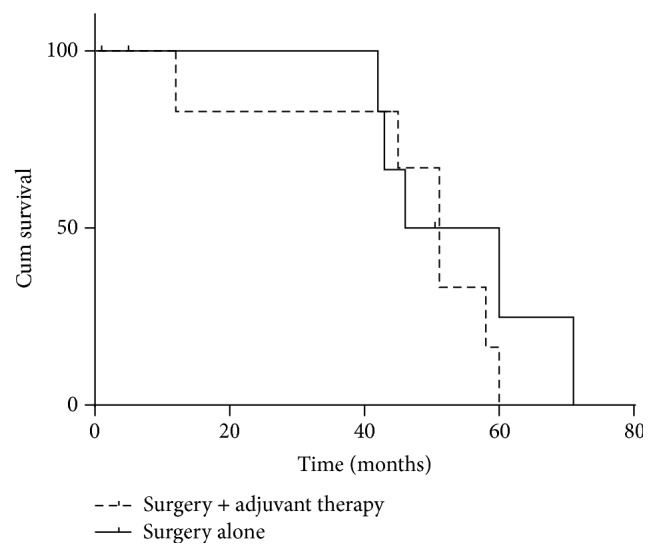
Survival curves of surgery alone group and surgery plus adjuvant therapy group.

In terms of the depth of invasion, T2, T3, T4a, and T4b occurred in 1 (6.7%), 9 (60%), 3 (20%), and 2 (13.3%) patients, respectively, regional lymph nodes metastases of N1, N2, and N3 occurred in 3 (20%), 1 (6.7%), and 3 (20%) cases, respectively, and stages IB, IIA, IIB, IIIA, IIIB, IIIC, and IV were detected in 1 (6.7%), 5 (33.3%), 2 (13.3%), 1 (6.7%), 3 (20%), 2 (13.3%), and 1 (6.7%) patients, respectively. After surgery, eight patients received no further treatment, seven had cisplatin-based or 5-fluorouracil-based adjuvant chemotherapy, including FP (5-fluorouracil + cisplatin), TP (paclitaxel + cisplatin), Tegafur Gimeracil Oteracil Potassium Capsule (S-1), and SOX (S-1 + Oxaliplatin), XELOX (Xeloda + Oxaliplatin), and among the seven patients, one patient also received adjuvant radiotherapy (PTV = 50.4 Gy/28f). Of the fifteen patients who underwent curative surgery (radical resection and D2 lymphadenectomy), eleven patients were alive and 4 patients died with a survival time of 1–50 months when the follow-up was ended, among which 2 patients died from haematemesis and the other 2 patients died from progressive disease. The patient who received FP chemotherapy regimen survived with a survival time of 45 months when the follow-up was ended. No significant difference in median survival time was observed between the surgery alone group and the surgery plus adjuvant therapy group (46 versus 51 months, *P* = 0.310, Figure 3).

### 3.6. The Nonsurgery Group

There were six patients in this group; four were diagnosed with metastatic disease by CT scan; among them, four were with liver metastasis, one was with spleen metastasis, and one was with adrenal gland metastasis; three of the 6 patients (numbers 1, 4, and 20) were treated with palliative chemotherapy; among them patient number 1 received 2 cycles of SOX at first and got efficacy evaluation of stable disease (SD) after contrast-enhanced computed tomography scan, and then the patient received a cycle of EOS (Epirubicin + Oxaliplatin + S-1) but died from haematemesis soon; patient number 4 received a cycle of DS (Docetaxel + S-1), four degrees of bone marrow suppression after chemotherapy occurred, and the patient died soon; patient number 20 received 2 cycles of chemotherapy, but the regimen was unknown and they died from progressive disease (PD), while the rest of three refused to receive any treatment. In terms of the depth of invasion, T3, T4a, and T4b occurred in 1 (16.7%), 3 (50%), and 2 (33.3%) patients, respectively; regional lymph nodes metastases of N1 and N2 occurred in 1 (16.7%) and 2 (33.3%) cases, respectively; stages IIA, IIB, and IV were detected in 1 (16.7%), 1 (16.7%), and 4 (66.6%) patients, respectively.

## 4. Discussion

Although gastric carcinoma remains one of the commonest gastrointestinal malignancies, the PGSCC is extremely rare, about 0.04% to 0.07% among other gastric cancers [1, 2]. It occurs mostly in men and the male/female ratio is 5 to 1, the peak incidence is in the sixth decade [4–6], and the primary lesions were mostly found in the upper third of the stomach [4]. The characteristics of the present cases are typical of those of past cases with regard to the gender, age at the time of diagnosis, and the location of carcinoma. In this study, 13 patients (61.9%) had long history of smoking and according to our observation, like lung squamous carcinoma and esophageal squamous carcinoma, long history of smoking may promote the occurrence of this tumor.

Five main theories regarding the origin of SCC in the stomach were proposed by Straus et al. [2, 6]: (1) squamous differentiation in a preexisting adenocarcinoma; (2) squamous metaplasia of the gastric mucosa before malignant transformation; (3) multipotential stem cells in the gastric mucosa which can develop into any type of cell; (4) nests of ectopic squamous cells in gastric mucosa; and (5) SCC arising from the vascular endothelium of the stomach. We particularly support the first hypothesis, because the hypothesis was supported by a report of three PGSCC tumors which were reexamined and areas of adenocarcinoma were found; furthermore, in our study, adenocarcinoma-related antigen such as CA19-9 was found in 7 (33.3%) cases of 21 patients with PGSCC. In addition to the five main hypotheses, Takita et al. [7] proposed that Epstein-Barr virus infection may be involved in the pathogenesis of at least some gastric SCC; however, there was no evidence of EBV infection in our patients.

The Japanese Gastric Cancer Association proposed the following criteria for the diagnosis of PGSCC [8]: (1) all the tumor cells are SCC cells and any part does not contain gland cancer cells; (2) there is sufficient evidence to show that SCC originates in the gastric mucosa. Our 21 cases were compatible with these criteria and thus were diagnosed as PGSCCs. Once PGSCC has been diagnosed, at least one of the following four histopathological criteria as proposed by Boswell and Helwig [9], permit the confirmation of the diagnosis: keratinized cell masses forming keratin pearls, a mosaic cell arrangement, intracellular bridges, and high concentrations of sulphydryl or disulfide groups. Our patients' histopathological findings were consistent with these criteria. Some immunohistochemistry studies have found strong staining for p63 and high-molecular-weight cytokeratin (CK5/6) with a specificity of 99% and a sensitivity of 98% for squamous cell carcinoma [5, 10]; we had four patients who underwent immunohistochemical examination; the tumor cells had positive immunoreactivity for p63 and CK5/6, supporting the diagnosis of SCC.

As shown in our results, more than half of the PGSCC cases were already relatively advanced at the time of diagnosis and had progressed to at least stage III (52.4%). Patients with PGSCC have variable clinical symptoms, such as abdominal pain, dysphagia, nausea and vomiting, melena or hematochezia, haematemesis, and weight loss, most of which are identical to those of other types of gastric tumors. Raju et al. [11] reported a case of a patient with squamous cell carcinoma of the stomach and hypercalcaemia; in their opinion, the hypercalcaemia of malignancy is due to ectopic production of parathyroid hormone (PTH) by the tumor; however, there was no hypercalcaemia in our patients; on the contrary, we observed hypocalcemia in nine patients; in addition, we also observed hypoalbuminemia in 9 patients; we hypothesized that patients with hypocalcemia or hypoalbuminemia were related to inadequate intake and excessive consumption of the disease. Because of the limited number of cases, we did not find whether hypocalcemia and hypoalbuminemia were associated with prognosis or not. Needless to say, the development of sophisticated detection methods can contribute to setting up early diagnosis of PGSCC; early diagnosis is most important to improve the prognosis of PGSCC.

Our study found that the prognosis of surgery or not was different; surgery to achieve R0 (no residual tumor) resection remains the mainstay of the treatment. A standard chemotherapy regimen for this disease has not yet been established; the choice of its treatment tends to follow the principle of the treatment of gastric adenocarcinoma. 5-Fluorouracil-based chemotherapy regimens were commonly used in the previous cases. In our study, the chemotherapy regimens of PGSCC are similar to the current chemotherapy regimens of gastric adenocarcinoma. For our patients, three received palliative chemotherapy, and seven received adjuvant chemotherapy. Nevertheless, there was no significant difference in median survival time observed between the surgery alone group and the surgery plus adjuvant therapy group (46 versus 51 months, *P* = 0.310) in the present study, but we found that patients who received adjuvant chemotherapy tended to prolong survival time, probably because the number of our cases is too small and so did not show statistical difference. Our 21 patients did not receive neoadjuvant therapy before surgery; Marubashi et al. [12] have demonstrated the efficacy of chemotherapy against the tumor; the patient was given neoadjuvant chemotherapy of low dose FP, demonstrating striking effectiveness of chemotherapy in the neoplasm both radiologically and histologically; no side effect occurred and the patient completed all the cycles of chemotherapy without any complication. However, not much information is available on the role of neoadjuvant chemoradiotherapy in gastric SCC, in comparison with other upper aerodigestive tumors like esophageal SCC where it has been shown to have a definite role.

Some studies reported that PGSCC has a better prognosis than gastric adenocarcinoma [1, 13]. However, PGSCC generally had poor outcomes because it is usually diagnosed at an advanced stage with marked infiltrative growth and aggressively metastasizes to the liver, the lymph nodes, and other organs [14–17]. We need further research in this tumor and further studies would benefit the patients affected by this rare disease.

## 5. Conclusions

PGSCC is extremely rare, it occurs mostly in men, the peak incidence is in the sixth decade, and the primary lesions are mostly found in the upper third of the stomach. Most of the clinical manifestations are identical to those of other types of gastric tumors; the tumor cells have positive immunoreactivity for p63 and CK5/6. In terms of treatments, surgery (R0 resection) is the main treatment; the effect of other treatments is unclear. The choice of its chemotherapy regimens tends to follow the principle of the treatment of gastric adenocarcinoma or esophageal cancer. PGSCC generally had a poor prognosis; early detection, early diagnosis, and early surgical treatment are beneficial to patients.

## Figures and Tables

**Table 1:** Clinicopathological characteristics and demographical data of 21 patients with PGSCC.

Number	Gender	Age	PS score	Site	Size (cm)	Borrmann type	HGB^a^	ALB^b^	Ca^2+c^
1	m	48	1	Gastric body (M)	5.8	I	51	32	2.02
2	f	48	1	Gastric body (M)	15	I	102	33.6	2.17
3	f	72	1	Gastric antrum (L)	5	II	141	35	1.98
4	m	52	1	Gastric body (M)	6	III	87	26.5	1.87
5	m	61	3	Gastric body (M)	5.6	I	58	28.9	1.89
6	m	67	3	Fundus of stomach (U)	7.3	I	87	36.5	2.05
7	m	61	0	Fundus of stomach (U)	8	I	82	28.7	2.04
8	m	59	1	Fundus of stomach (U)	5	I	90	41.3	2.16
9	m	68	1	Fundus of stomach (U)	8	II	118	41.1	2.25
10	f	67	2	Fundus of stomach (U)	9	III	118	36.9	1.88
11	m	75	0	Gastric cardia (U)	4	III	123	41.1	2.14
12	m	68	1	Gastric cardia (U)	5	II	167	43.3	2.29
13	m	63	0	Gastric body (M)	4	II	127	40	2.19
14	m	67	1	Gastric body (U)	8	I	90	35.8	2.20
15	m	67	1	Gastric cardia (U)	4.5	II	110	31.5	1.93
16	m	67	1	Gastric cardia (U)	7	II	112	34.7	2.22
17	m	48	1	Gastric cardia (U)	3	I	135	39.9	2.16
18	m	65	1	Gastric cardia (U)	3	I	111	43.7	2.17
19	m	69	1	Gastric cardia (U)	8	III	150	27.8	2.00
20	m	73	2	Gastric body (M)	9.5	I	109	33.8	2.10
21	m	55	1	Fundus of stomach (U)	4	III	152	42.1	2.27

PGSCC: primary gastric squamous cell carcinoma; m: male; f: female; PS score: performance status score; U: upper third of stomach; M: middle third of stomach; L: lower third of stomach; ^a^normal range: 120–160 g/L; ^b^normal range: 35–55 g/L; ^c^normal range: 2.1–2.7 mmol/L.

**Table 2:** Clinicopathological characteristics and survival outcomes of 21 patients with PGSCC.

Number	TNM stage	Depth of invasion	Metastasis of regional LN	Distant metastasis	Treatment	Outcomes (months)
1	IV	T4b	N2	M1	P	Death (6)
2	IIIB	T4b	N0	M0	R + A	Death (5)
3	IIB	T3	N1	M0	R + A	Alive (12)
4	IV	T4a	N2	M1	P	Death (1)
5	IV	T4b	N1	M1	No	Death (2)
6	IIA	T3	N0	M0	No	Death (5)
7	IIA	T3	N0	M0	R + A	Alive (60)
8	IIA	T3	N0	M0	R + A	Alive (45)
9	IIIC	T4b	N3	M0	R	Death (1)
10	IIB	T4a	N0	M0	No	Death (4)
11	IIIC	T4a	N3	M0	R	Death (50)
12	IB	T2	N0	M0	R	Alive (60)
13	IIIB	T4a	N2	M0	R	Alive (71)
14	IIA	T3	N0	M0	R + A	Alive (58)
15	IIA	T3	N0	M0	R + A	Alive (51)
16	IV	T3	N0	M1	R + A	Alive (51)
17	IIIA	T4a	N1	M0	R	Alive (46)
18	IIA	T3	N0	M0	R	Alive(43)
19	IIIB	T3	N3	M0	R	Alive (42)
20	IV	T4a	N0	M1	P	Death (6)
21	IIB	T3	N1	M0	R	Death (19)

PGSCC: primary gastric squamous cell carcinoma; LN: lymph nodes; P: palliative therapy; R: radical operation; A: adjuvant therapy.

## References

[1] Bonnheim D. C., Sarac O. K., Fett W. (1985). Primary squamous cell carcinoma of the stomach. *The American Journal of Gastroenterology*.

[2] Straus R., Heschel S., Fortmann D. J. (1969). Primary adenosquamous carcinoma of the stomach. A case report and review. *Cancer*.

[3] Washington K. (2010). 7th edition of the AJCC cancer staging manual: stomach. *Annals of Surgical Oncology*.

[4] Wakabayashi H., Matsutani T., Fujita I. (2014). A rare case of primary squamous cell carcinoma of the stomach and a review of the 56 cases reported in Japan. *Journal of Gastric Cancer*.

[5] Callacondo-Riva D., Ganoza-Salas A., Anicama-Lima W., Quispe-Mauricio A., Longacre T. A. (2009). Primary squamous cell carcinoma of the stomach with paraneoplastic leukocytosis: a case report and review of literature. *Human Pathology*.

[6] Schmidt C., Schmid A., Lüttges J. E., Kremer B., Henne-Bruns D. (2001). Primary squamous cell carcinoma of the stomach. Report of a case and review of literature. *Hepato-Gastroenterology*.

[7] Takita J., Kato H., Miyazaki T. (2005). Primary squamous cell carcinoma of the stomach: a case report with immunohistochemical and molecular biologic studies. *Hepato-Gastroenterology*.

[8] Japanese Gastric Cancer Association (2011). Japanese classification of gastric carcinoma: 3rd English edition. *Gastric Cancer*.

[9] Boswell J. T., Helwig E. B. (1965). Squamous cell carcinoma and adenoacanthoma of the stomach. A clinicopathologic study. *Cancer*.

[10] von Waagner W., Wang Z., Picon A. I. (2015). A rare case of a primary squamous cell carcinoma of the stomach presenting as a submucosal mass. *Case Reports in Surgery*.

[11] Raju G. C., Barton E. N., Marchack D., Naraynsingh V. (1987). Hypercalcaemia in primary squamous cell carcinoma of the stomach. *Journal of the Royal Society of Medicine*.

[12] Marubashi S., Yano H., Monden T. (1999). Primary squamous cell carcinoma of the stomach. *Gastric Cancer*.

[13] Altshuler J. H., Shaka J. A. (1966). Squamous cell carcinoma of the stomach. Review of the literature and report of a case. *Cancer*.

[14] Dursun M., Yaldiz M., Isikdogan A. (2003). Primary squamous cell carcinoma of the stomach: a case report and review of the literature. *European Journal of Gastroenterology and Hepatology*.

[15] Muto M., Hasebe T., Muro K. (1999). Primary squamous cell carcinoma of the stomach: a case report with a review of Japanese and Western literature. *Hepato-Gastroenterology*.

[16] Volpe C. M., Hameer H. R., Masetti P., Pell M., Shaposhnikov Y. D., Doerr R. J. (1995). Squamous cell carcinoma of the stomach. *The American Surgeon*.

[17] Mori M., Iwashita A., Enjoji M. (1986). Squamous cell carcinoma of the stomach: report of three cases. *American Journal of Gastroenterology*.

